# Estimation of incubation period distribution of COVID-19 using disease onset forward time: A novel cross-sectional and forward follow-up study

**DOI:** 10.1126/sciadv.abc1202

**Published:** 2020-08-14

**Authors:** Jing Qin, Chong You, Qiushi Lin, Taojun Hu, Shicheng Yu, Xiao-Hua Zhou

**Affiliations:** 1 Biostatistics Research Branch, National Institute of Allergy and Infectious Diseases, National Institutes of Health, Rockville, MD 20852, USA.; 2 Beijing International Center for Mathematical Research, Peking University, 100871, China.; 3 School of Mathematical Sciences, Peking University, 100871, China.; 4 Office for Epidemiology, Chinese Center for Disease Control and Prevention, 102206, China.; 5 Department of Biostatistics, School of Public Health, Peking University, 100871, China.; 6 Center for Statistical Science, Peking University, 100871, China.

## Abstract

We have proposed a novel, accurate low-cost method to estimate the incubation-period distribution of COVID-19 by conducting a cross-sectional and forward follow-up study. We identified those presymptomatic individuals at their time of departure from Wuhan and followed them until the development of symptoms. The renewal process was adopted by considering the incubation period as a renewal and the duration between departure and symptoms onset as a forward time. Such a method enhances the accuracy of estimation by reducing recall bias and using the readily available data. The estimated median incubation period was 7.76 days [95% confidence interval (CI): 7.02 to 8.53], and the 90th percentile was 14.28 days (95% CI: 13.64 to 14.90). By including the possibility that a small portion of patients may contract the disease on their way out of Wuhan, the estimated probability that the incubation period is longer than 14 days was between 5 and 10%.

## Teaser

This study estimated the incubation distribution of COVID-19 using the theory of renewal process.

## INTRODUCTION

The Center for Disease Control and Prevention (CDC) of China and World Health Organization are closely monitoring the current outbreak of coronavirus disease 2019 (COVID-19). As of 22 February 2020, the National Health Commission of China had confirmed a total of 76,936 cases of COVID-19 in mainland China, including 2442 fatalities and 22,888 recoveries (*1*). Various containment measures, including travel restrictions, isolation, and quarantine have been implemented in China with the aim of minimizing virus transmission via human-to-human contact (*2*). Quarantine of individuals with exposure to infectious pathogens has always been an effective approach for containing contagious diseases in the past. One of the critical factors to determine the optimal quarantine of presymptomatic individuals is a good understanding of the incubation period, and this has been lacking for COVID-19.

The incubation period of an infectious disease is the time elapsed between infection and appearance of the first symptoms and signs of disease. Precise knowledge of the incubation period would help to provide an optimal length of quarantine period for disease control purpose and also is essential in the investigation of the mechanism of transmission and development of treatment. For example, the distribution of the incubation period is used to estimate the reproductive number *R*, that is, the average number of secondary infections produced by a primary case. The reproductive number is a key quantity that affects the potential size of an epidemic. Despite the importance of the incubation period, it is often poorly estimated on the basis of limited data.

To the best of our knowledge, there is only a handful of studies estimating the incubation period of COVID-19. Among them are Li *et al*. (*3*), Zhang *et al*. (*4*), Guan *et al*. (*5*), Backer *et al*. (*6*), Linton *et al*. (*7*), and Lauer *et al.* (*8*). The estimates of the incubation period from these five studies, together with other results of two other coronavirus disease, severe acute respiratory syndrome (SARS) and Middle East respiratory syndrome (MERS), are listed in Table 1. In Li *et al.* (*3*), the first 425 lab-confirmed cases, reported as of 22 January 2020, were included in the study, but the exact dates of exposure could be identified in only 10 of these cases. The distribution of the incubation period was subsequently approximated by fitting a lognormal distribution to these 10 data points, resulting in a mean incubation period of 5.2 days [95% confidence interval (CI): 4.1 to 7.0], and the 95th percentile is 12.5 days. Similarly, in Zhang *et al.* (*4*), 49 cases with no travel history who were identified by prospective contact tracing were used to estimate incubation period by fitting a lognormal distribution, resulting in a mean incubation period of 5.2 days (1.8 to 12.4). However, given the limited sample size, it is challenging to make a solid inference on the distribution of the incubation period. A different result was reported by Guan *et al*. (*5*), based on 291 patients who had clear information regarding the specific date of exposure as of 29 January 2020, stating that the median incubation period was 4.0 days (interquartile range, 2 to 7). However, this study of the incubation period can be highly influenced by the individuals’ recall bias or interviewers’ judgment on the possible dates of exposure rather than the actual dates of exposure that, in turn, might not be accurately monitored and determined, thus leading to a high percentage of error. In Backer *et al*. (*6*), 88 confirmed cases detected outside Wuhan were used to estimate the distribution of the incubation period. For each selected case, a right-censored observation of the incubation period can be obtained by travel history and symptoms onset. The distribution of the incubation period can then be estimated by fitting a Weibull, Gamma, or lognormal distribution with censored data. However, this method contained two types of sampling biases: (i) With the longer incubation period, the patients who resided at Wuhan but developed symptoms outside Wuhan were easier to be observed (i.e., a patient with a shorter incubation period would develop symptoms before the planned trip and possibly cancel the trip; hence, such case would not be observed) and, therefore, lead to an overestimation; (ii) if the follow-up time (from infection to the end of the study) is short, then only the shorter incubation period would be observed and hence lead to an underestimation (i.e., assume information of confirmed cases from days 1 to 10 was collected, two patients, A and B, both got infected on the day 5, patient A had an incubation period of 2 days while patient B had an incubation period of 8 days, then only patient A with the shorter incubation period would be included in the data, patient B with the longer incubation period would develop symptoms after day 10 hence would not be included in the data). Linton *et al*. (*7*) proposed a similar approach to the study of Backer *et al*. (*6*) with a larger sample size of 152 but, in addition, corrected the second sampling bias aforementioned. However, the first problem in regard to the sampling bias is still an unsolved issue. In Lauer *et al.* (*8*), a pooled data with sample size of 181 were used to estimate the incubation period. All collected cases in the pooled data had identifiable exposure and symptom onset windows available, of which 161 had a known recent history of travel to or residence in Wuhan, which was the same kind of data collected in Backer *et al*. (*6*) and Linton *et al*. (*7*); others had evidence of contact with travelers from Hubei or persons with known infection. A similar approach to Backer *et al*. (*6*) was used, and the aforementioned two issues in regard to sampling bias remain unsolved. Lauer *et al*. (*8*) reported that 2.5% of patients developed symptoms after 11.5 days and claimed that it was highly unlikely that further symptomatic infections would be undetected after 14 days, while the same co-authors reported 5% of patients have symptoms onset after 14 days in the study of Bi *et al.* (*9*).

**Table 1:** Estimates for the incubation periods of SARS, MERS, and COVID-19. NA, not available.

**Incubation** **distribution** **metric**	**SARS**	**MERS**	**COVID-19**
Mean (SD) or mean (95% CI)	Hong Kong (*14*): 4.4 (4.6)	Saudi Arabia (*15*): 5.0 (4.0–6.6)	Wuhan (*3*): 5.2 (4.1–7.0)
	Beijing (*14*): 5.7 (9.7)	South Korea (*15*): 6.9 (6.3 to 7.5)	Mainland China (*4*): 5.2 (1.8–12.4)
	Taiwan (*14*): 6.9 (6.1)	South Korea (*16*): 6.9 (6.3 to 7.5)	Mainland China (*6*): 5.8 (4.6–7.9)
	Hong Kong (*17*): 4.6 (15.9)	Saudi Arabia (*16*): 5.0 (4.4 to 6.6)	Global (*7*): 5.6 (5.0–6.3)
	Mainland China (*20*): 5.29 (12.33)	South Korea (*19*): 6.7 (6.1 to 7.3)	Global (*8*): 5.5
	Singapore (*21*): 4.83 (4.37–5.29)		
	Hong Kong (*21*): 6.37 (5.29–7.75)		
Median or median (95% CI)	Hong Kong, Canada, and USA (*22*): 4	South Korea (*19*): 6.0 (4.0–7.0)	Mainland China (*5*): 3.0
		Middle East (*23*): 5.2 (1.9–14.7)	Global (*8*): 5.1 (4.5–5.8)
		South Korea (*24*): 6.3 (5.7–6.8)	
Percentiles	Mainland China, 90% (*18*): 10.7	NA	Mainland China, 2.5% (*6*): 1.3
	Hong Kong, Canada, and USA, 90% (*22*): 12		Wuhan, 95% (*3*): 12.5
	Singapore, 95% (*20*): 9.66 (0.5)		Mainland China, 97.5% (*6*): 11.3
	Mainland China, 95% (*18*): 13.91		Global, 2.5% (*8*): 2.2 (1.8–2.9)
	Hong Kong, 95% (*21*): 14.22		Global, 97.5% (*8*): 11.5 (8.2–15.6)
	Mainland China, 99% (*18*): 20.08		

To overcome the aforementioned problems, we propose a novel method to estimate the incubation period of COVID-19 by using the well-known renewal theory in probability (*10*). Such a method enhances the accuracy of estimation by reducing recall bias and using abundance of the readily available forward time with a large sample size of 1084. To the best of our knowledge, our study of the distribution of the incubation period involves the largest number of samples to date. We find that the estimated median of the incubation period is 7.76 days (95% CI: 7.02 to 8.53), mean is 8.29 days (95% CI: 7.67 to 8.9), the 90th percentile is 14.28 days (95% CI: 13.64 to 14.90), and the 99th percentile is 20.31 days (95% CI: 19.15 to 21.47). Furthermore, by including the possibility that a small portion of patients may contract the disease on their way out of Wuhan, the estimated tail probability that incubation period is longer than 14 days is between 5 and 10%. It is difficult to estimate the proportion of incubation beyond 14 days in general if the sample size is small. Because our sample size is much larger than that of other studies published to date, we have confidence in the robustness of our findings. Our estimated incubation period of COVID-19 is longer than those given by previous researches on SARS, MERS, and COVID-19 in Table 1.

## METHODS

### Motivations

As described in the previous section, the distribution of the incubation period in most of the literature is either described through a parametric model or its empirical distribution based on the observed incubation period from the contact tracing data. However, the contact tracing data are challenging and expensive to obtain, and their accuracy can be highly influenced by recall bias. Hence, a low-cost and high-accuracy method to estimate the incubation distribution is needed. In this study, we make use of confirmed cases detected outside Wuhan with known histories of travel or residency in Wuhan to estimate the distribution of incubation times. The renewal theory is implemented by treating an incubation period of a prevalence case as a renewal process. See more details of the renewal process and corresponding assumptions in section S1.

### Data collection and justification

Publicly available data were retrieved from provincial and municipal health commissions in China and the ministries of health in other countries, including 12,963 confirmed cases outside Hubei Province as of 15 February 2020. Detailed information on confirmed cases includes region, gender, age, date of symptom onset, date of confirmation, history of travel or residency in Wuhan, and date of departure from Wuhan. The date of symptoms onset in these data refers to the date reported by the patient on which the clinical symptoms first appeared, where the clinical symptoms include fever, cough, nausea, vomiting, diarrhea, and others. Among 12,963 confirmed cases, 6345 cases had their dates of symptom onset collected, 3169 cases had histories of travel or residency in Wuhan, 2514 cases had their dates of departure recorded, and 1922 cases had records of both dates of departure from Wuhan and dates of symptoms onset. However, not all 1922 cases should be taken in the analysis. After examining the collected data, there were a total of 1084 cases that meet the criteria described in section S2 and were followed forwardly.

**Fig. 1:**
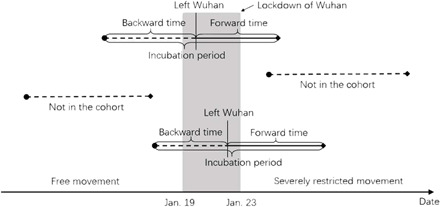
Illustration of our cross-sectional and forward follow-up study. Backward and incubation periods are not observed, while Wuhan departure and forward time are observed.

Figure 1 shows the design of the cross-sectional and forward follow-up study. The dot on the left end of each segment is a date of infection, while the square on the right end is a date of symptoms onset. The date of departure from Wuhan cuts the line segment in between. Note that only solid lines were followed in our cohort, while dashed lines are not followed in the cohort because the date of departure from Wuhan is not between 19 January 2020 and 23 January 2020.

Among the 1084 cases with gender information in the study, 468 (43.30%) are female. The mean age of patients was 41.31, and the median age was 40. More than 80% of the cases were between 20 and 60. The youngest confirmed case in our cohort was 6 months old, while the oldest was 86 years old. Table 2 shows the demographic characteristics of patients with COVID-19 in the Wuhan departure cohort and the entire data collected as of 15 February 2020. Although there are slight differences between the selected cases and all cases, we explored the correlation between forward time and age instead and found that the correlation between forward time and age was −0.0309. Hence, there is no evidence that the incubation time depends on age in this dataset, and the observed forward times should be able to represent that of in the general population. More demographic characteristics of patients are summarized in section S2.

**Table 2:** Comparison between the demographic characteristics of patients with COVID-19 in the studying cohort and all publicly available cases collected as of 15 February 2020.

**Age group (years)**	**Female**		**Male**		**No information**	
	**Study cohort**	**All cases**	**Study cohort**	**All cases**	**Study cohort**	**All cases**
	468 (43.3)*	**4121 (47.3)**	**614 (57.1)**	**4597 (52.7)**	**2**	**4245**
0–19	17 (3.7)	126 (3.2)	24 (4.0)	180 (4.2)	0	3
20–39	189 (40.9)	1250 (32.2)	292 (48.3)	1508 (35.0)	1	48
40–59	195 (42.2)	1667 (43.0)	226 (37.4)	1843 (42.8)	0	57
60–79	60 (13.0)	749 (19.3)	62 (10.2)	701 (16.3)	0	40
≥80	1 (0.2)	85 (2.2)	1 (0.2)	78 (1.8)	0	8
No information	6	244	9	287	0	4089

*Number (%). The percentages do not take missing data into account.

### Estimation of incubation period distribution of COVID-19

Let *Y* be the incubation period of an infected case with probability density function *f*(*y*) where *y* > 0. Let *A* be the duration from infection in Wuhan to the departure of Wuhan, which can be considered as the backward time in a renewal process. Let *V* denote the duration between the departure from Wuhan and the onset of symptoms, which can be considered as the forward time in a renewal process. Then, *V* has the density as follows

$$g(v)=\frac{\bar{F}(v)}{\mu },v\geq 0$$ (1)

where $\bar{F}(∙)$ is the survival function corresponding to *f*( ∙ ), and $\mu =\int_{0}^{\infty }\mathrm{yf}(y)\mathrm{dy}$ is the mean incubation period. Note that *A* and *V* have the same density marginally, and the aforementioned sampling bias can be corrected by using Eq. 1. See more technical details in section S3.

In our cohort of COVID-19 cases, we assume that the incubation period is a Weibull random variable; the estimates in the Weibull model can be obtained by maximizing the corresponding likelihood function. The mean and percentiles of the incubation period can be calculated from the parametric Weibull distribution. The CIs in this study are obtained using bootstrap method with *B* = 1000 resamples. Note that Gamma distribution and lognormal distribution are also fitted for the incubation, both provide similar estimates of quantiles compared with Weibull.

### Sensitivity analysis

It is arguable that people who left Wuhan might also be infected on the day of departure since they had a higher chance to be exposed to this highly contagious, human-to-human–transmitted virus in a crowded environment, as cases were increasing. In this case, the duration between departure from Wuhan and onset of symptoms is no longer only the forward time but a mixture of the incubation period and the forward time. Unfortunately, it is unclear who got infected before departure and who got infected at the event of departure. Hence, a mixture sensitivity forward time model is proposed, that is

$$h(v)=\alpha \lambda \{\pi {\left(v\lambda \right)}^{\alpha -1}+(1-\pi )/\Gamma (1/\alpha )\}\text{exp}(-{\left(v\lambda \right)}^{\alpha }),v\geq 0$$ (2)

If α ≠ 1, then it is possible to identify all underlying parameters. We explore the sensitivity of estimates of incubation period by assuming a range of π, that is, π = 0,0.05,0.1, and 0.2 and estimate α and λ by maximizing the product of likelihoods, $\prod_{i=1}^{I}h(v_{i})$, with respect to α and λ, where *v_i_* is the observed forward time of the *i*th individual and *I* is the sample size of the studying cohort.

## RESULTS

**Fig. 2:**
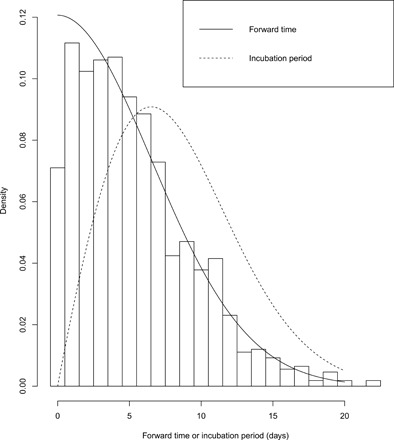
Histogram and estimated probability density functions for the time from Wuhan departure to symptoms onset, i.e., forward time.

By fitting the observed forward times *v_i_* of the 1084 cases in our cohort to the likelihood function (Eq. 2), we find that π = 0 gives the largest log likelihood; hence, we set π = 0 as the reference scenario. The maximum likelihood estimates are $\hat{\alpha }=1\cdot 97$ (95% CI: 1.75 to 2.28) and $\hat{\lambda }=0.11$ (95% CI: 0.10 to 0.12) in our reference scenario. The estimated 5th, 25th, 50th, 75th, 90th, 95th, 99th, and 99.9th percentiles of the incubation period are 2.07 (95% CI: 1.60 to 2.69), 4.97 (95% CI: 4.25 to 5.78), 7.76 (95% CI: 7.02 to 8.53), 11.04 (95% CI: 10.34 to 11.66), 14.28 (95% CI: 13.64 to 14.90), 16.32 (95% CI: 15.62 to 17.04), 20.31 (95% CI: 19.15 to 21.47), and 24.95 (95% CI: 23.04 to 26.81) days, respectively. The mean incubation period is 8.29 (95% CI: 7.67 to 8.90) days. Estimates based on Gamma distribution and lognormal distribution provide very similar results, where the 50th percentile is 8.16 and 8.42, respectively, the 90th percentile is 14.23 and 14.11, respectively, and the log likelihoods are −2843.34 and −2845.57, which are slightly smaller compared with the Weibull distribution. The average time from leaving Wuhan to the symptom onset is 5.30 days, the sample median is 5 days, and the maximum is 22 days. Figure 2 visualizes the fitted density function in Eq. 2 in a solid line onto the histogram of observed forward times, and the dashed line is the Weibull probability density function for incubation period distribution. Note that Eq. 2 fits the observed forward times well, suggesting that our model is reasonable and the results are therefore trustworthy.

Table 3 summarizes the estimates of the parameters and the mean and percentiles of the incubation period. We can see that the estimates for mean and percentiles decrease as the proportion of people who got infected at the event of departure, π, increases. However, variation of the results from π = 0 to 0.2 is only about 1 day, which we believe is still in an acceptable range.

**Table 3:** Results of our model based on different choices of π.

**Scenario**	**Reference** **case**	**Additional % infected on the Wuhan** **departure day**		
	**π = 0**	**π = 5%**	**π = 10%**	**π = 20%**
$\hat{\alpha }$	1.97 (1.75, 2.28)	1.93 (1.72, 2.22)	1.89 (1.69, 2.12)	1.81 (1.66, 2.02)
$\hat{\lambda }$	0.11 (0.1, 0.12)	0.11 (0.1, 0.12)	0.11 (0.11, 0.12)	0.12 (0.11, 0.13)
Mean	8.29 (7.67, 8.9)	8.01 (7.45, 8.61)	7.75 (7.23, 8.31)	7.32 (6.85, 7.8)
5%	2.07 (1.6, 2.69)	1.93 (1.5, 2.52)	1.81 (1.42, 2.3)	1.60 (1.29, 2)
25%	4.97 (4.25, 5.78)	4.73 (4.07, 5.49)	4.51 (3.92, 5.19)	4.14 (3.66, 4.7)
Median	7.76 (7.02, 8.53)	7.47 (6.78, 8.18)	7.19 (6.55, 7.9)	6.73 (6.19, 7.3)
75%	11.04 (10.34, 11.66)	10.7 (10.07, 11.35)	10.38 (9.78, 10.98)	9.86 (9.3, 10.4)
90%	14.28 (13.64, 14.9)	13.92 (13.32, 14.57)	13.59 (12.99, 14.17)	13.04 (12.44, 13.59)
95%	16.32 (15.62, 17.04)	15.95 (15.3, 16.65)	15.62 (14.91, 16.26)	15.07 (14.38, 15.72)
99%	20.31 (19.15, 21.47)	19.94 (18.87, 20.98)	19.62 (18.52, 20.62)	19.1 (17.98, 20.11)
99.9%	24.95 (23.04, 26.81)	24.6 (22.78, 26.31)	24.33 (22.65, 26.03)	23.89 (22.05, 25.43)
−Log likelihood	2843.00 (2796.63, 2889.72)	2843.21 (2799.86, 2891.41)	2843.57 (2795.53, 2887.36)	2844.96 (2796.74, 2890.19)

## DISCUSSION

A sound estimate of the distribution of the incubation period plays a vital role in epidemiology. Its application includes decisions regarding the length of quarantine for prevention and control, dynamic models that accurately predict the disease process, and determining the contaminated source in foodborne outbreaks. Here, we propose a novel method to estimate the incubation distribution that only requires information on travel histories and dates of symptoms onset. This method enhances the accuracy of estimation by reducing recall bias and using abundance of the readily available forward time data. To the best of our knowledge, this study of incubation period involves the largest number of samples to date. In addition, this is the first article to consider the incubation period for COVID-19 as a renewal process, which is a well-studied methodology and has a solid theoretical foundation. The estimated incubation period has a median of 7.76 days (95% CI: 7.02 to 8.53) and a mean of 8.29 days (95% CI: 7.67 to 8.90), the 90th percentile is 14.28 days (95% CI: 13.64 to 14.90), and the 99th percentile is 20.31 days (95% CI: 19.15 to 21.47). By including the possibility that a small portion of patients may contract the disease on their way out of Wuhan, the estimated tail probability that incubation period is longer than 14 days is between 5 and 10%. Compared with the results published in Li *et al*. (*3*), Guan *et al*. (*5*), Backer *et al*. (*6*), and Linton *et al*. (*7*), the incubation period estimated in our study is notably longer. Below is some evidence that may potentially support our findings of the long incubation period:

1) In the study of Guan *et al.* (*5*) on behalf of the China Medical Treatment Expert Group for COVID-19, the incubation period had a reported median of 4 days, the first quartile of 2 days, and the third quartile of 7 days. By fitting a commonly used Weibull distribution to these quartiles, we can obtain $\hat{\alpha }$ = 1.24 and $\hat{\lambda }=0.186$ defined in Eq. 2. As a consequence, the estimated 90, 95, and 99% percentiles are, respectively, 10.54, 13.04, and 18.45 days, which indicates that some patients may have extended incubation periods. In addition, in the commentary published in NEJMqianyan by the authors Guan *et al*. (*5*), it was reported that the incubation period of one patient in each of the severe and nonsevere groups was up to 24 days, 13 cases (12.7%) with an incubation period greater than 14 days and 8 cases (7.3%) with an incubation period greater than 18 days, which were close to what have found in our study (*11*).

2) One particular case reported by Yibin municipal health commissions in China stated that a 64-year-old female was diagnosed with COVID-19 on 11 February 2020 at Yibin, Sichuan Province 20 days after returning from Wuhan. This patient was under self-quarantine at home with the family for 18 days, from January 23 to February 9. On February 8, the patient developed mild symptoms of cough with sputum production (*12*).

3) It was reported in Bai *et al*. (*13*) that the incubation period for patient 1 was 19 days. However, the claimed 19-day incubation was the time difference between departure from Wuhan and symptoms onset, namely, the forward time in our study. The actual incubation period should be longer than 19 days.

On the basis of the estimated incubation distribution in this study, about 10% of patients with COVID-19 would develop symptoms after 14 days of infection. This may be a public health concern in regard to the current 14-day quarantine period. Our approach does require that certain assumptions are to be met, which we detail below.

1) The collection of forward time depends on the follow-up time, that is, if the follow-up time is not long enough, then we would only be able to include those with a shorter incubation period in the Wuhan departure cohort. This limitation may lead to an underestimation of the incubation period. The same limitation also applies to Backer *et al*. (*6*) and Linton *et al.* (*7*). However, as explained earlier, we only included cases who left Wuhan before January 23 in this study, which leaves an average follow-up time of 25 days. Hence, it is less likely that we missed those patients with longer incubation periods based on the largest incubation period of 24 days, as reported in Guan *et al*. (*5*). Note that the 24-day incubation period was reported as an outlier in Guan *et al*. (*5*).

2) We assume that the individuals included in our cohort were either infected in Wuhan or on the way to their destination from Wuhan, and violation of this assumption leads to an overestimation of incubation period. The same limitation also applies to Backer *et al*. (*6*), Linton *et al*. (*7*), and Lauer *et al*. (*8*). However, with a carefully selected cohort justified in Methods, the chance for an individual in the Wuhan departure cohort getting infected outside Wuhan should be relatively small. Nonetheless, we acknowledge that this possibility exists, for example, a family member could be uninfected by the time of departing Wuhan but got infected by other family members or outside contacts after leaving Wuhan. A sensitivity analysis was also conducted by removing all cases who left Wuhan with their families in the Wuhan departure cohort, and we found that it only resulted in a small change of the estimated distribution of the incubation period.

3) Individuals in our selected cohort were those who got infected in the early days of the outbreak. They were likely the first- or second-generation cases. Our results do not apply to higher generation cases if the virus mutates.

## Supplementary Material

abc1202_SM.pdf

## SUPPLEMENTARY MATERIALS

Supplementary material for this article is available at http://advances.sciencemag.org/cgi/content/full/6/33/eabc1202/DC1
