# Supplementary material for: Estimation of incubation period distribution of COVID-19 using disease onset forward time: A novel cross-sectional and forward follow-up study
Source: Sci Adv. 2020 Aug 14;6(33):eabc1202. doi: 10.1126/sciadv.abc1202 (PMC7428324; doi:10.1126/sciadv.abc1202)

Science Advances
AAAS

advances.sciencemag.org/cgi/content/full/sciadv.abc1202/DC1

Supplementary Materials for

Estimation of incubation period distribution of COVID-19 using disease onset forward time: a novel cross-sectional and forward follow-up study

Jing Qin, Chong You, Qiushi Lin, Taojun, Shicheng Yu, Xiao-Hua Zhou*

*Corresponding author. Email: azhou@math.pku.edu.cn

Published 7 August 2020, *Sci. Adv.* **6**, eabc1202 (2020)
DOI: 10.1126/sciadv.abc1202

This PDF file includes:

Sections S1 to S4
Table S1
Fig. S1

Supplementary Material

S1

Based on the COVID-19 daily updates from provincial and municipal health commissions in China, we notice that there is an abundance of cases who asymptomatically left Wuhan, the epicenter of COVID-19, and developed symptoms outside Wuhan. Assuming that these cases were infected before their departure from Wuhan, the time differences between departure and symptoms onset is the censored observations of their incubation periods. Hence, we conducted a cross-sectional and forward follow-up study by assuming to catch those asymptomatic individuals at their departure time and followed them until their symptoms developed. Using the language of renewal processes, we can treat the development of the disease starting from infection by a pathogen as a stochastic process that could be observed from a specific time point in chronological order. In this study, the specific time point refers to the time of departure from Wuhan. For each prevalent case, the complete process from the infection to the onset of symptoms can be considered as a renewal process. As illustrated in Figure 1, the backward recurrence time is hence defined as the time between infection and departure from Wuhan, and the forward recurrence time is the time between departure from Wuhan and symptom onset. Clearly, the forward time is observable and the corresponding observations are with good veracity, while the backward time is either unable to be observed or the corresponding observations are with large uncertainty due to recall bias. Note that for each infected individual, the backward time and forward time do not have to be same. However, when the renewal process reaches its equilibrium status, it becomes reversible, that is, the statistical properties of this process is the same as the one for time-reversed data in a same process. Hence, at equilibrium, the backward time can be treated as the forward time if time periods are reversed (25).

In order to model incubation using the renewal process properly, the following assumptions are established:

(A1). The renewal process has reached its equilibrium status;

(A2). The distribution of the incubation period is continuous;

(A3). The distribution of the incubation period has a finite first moment;

(A4). The incubation period for each case is independent and identically distributed;

(A5). The cases included in the analysis were infected at Wuhan and developed their symptoms outside Wuhan.

In this study, it is reasonable to assume (A1) is satisfied between January 19, 2020, and January 23, 2020, because there are over eleven million residents in the Wuhan metropolitan area and nearby neighborhoods and the daily travel volume in and out of Wuhan exceeded million before January 23, 2020. We justify

the use of data between January 19 and January 23 below. With adequate long run, the renewal process would reach the equilibrium status. The assumptions (A2) to (A4) are standard. In fact, we may assume that the incubation period is a continuous variable with range $(0, M)$ for some finite number M. It is well known that the first moment exists for a bounded random variable. The justification for assumption (A5) is below. Therefore, the probability renewal process theory can be applied with confidence, and thus we can avoid the challenging mission of ascertaining the backward time.

S2

As of February 15, 2020, 1922 cases had records of both dates of departure from Wuhan and dates of symptoms onset. However, not all 1922 cases should be taken in the analysis. We have to ensure that 1). assumption (A5) is satisfied, and 2). the follow-up time is long enough. Epidemiological information has indicated that about 90% of cases were directly imported from Wuhan before January 24, 2020 which partial supports (A5) (26).

To further make sure that the assumption (A5) is being satisfied as much as possible, we

(1) exclude cases whose first symptoms appeared before departure;
(2) exclude all cases who left Wuhan with their infected relatives and friends; and
(3) exclude cases who left Wuhan before January 19, 2020.

It is less likely that a case with travel or residency history at Wuhan was infected by a local case without visiting Wuhan. In fact the daily epidemic report produced by some local health commissions in China would detail if the local case had closed contact with cases imported from Wuhan and consider it as the most possible exposure to cause the infection. If a case with travel or residency history at Wuhan was indeed infected outside Wuhan (excluding the case who was infected on the way from Wuhan to its destination, such case would be considered separately in Sensitivity analysis in the manuscript), it is most likely to be infected by other imported cases from Wuhan. Considering the population density of people having travel history at Wuhan in other provinces, it was unlikely they got the virus from random imported cases from Wuhan other than their friends and relatives. Hence, by removing cases whose families and friends were also infected in our cohort, the probability of infection after departure is low. Furthermore, the date of January 19, 2020 was used because before January 19, the Chinese public was not aware of the severity of this epidemic, and those who left Wuhan might still have had close contact with other infected cases from Wuhan and hence actually got infected outside Wuhan. However, starting January 19, the China CDC began issuing test reagents to all provinces, confirmed cases were reported outside Hubei province in mainland China, the severity of COVID-19 was widely noted by the public,

and various unprecedented strict containment measures were implemented to minimize human-to-human transmission.[2] Thus, with all these three criteria, it is unlikely that confirmed cases who left Wuhan after January 19, 2020, were infected outside Wuhan and assumption (A5) is supported. Nonetheless, we do acknowledge the possibility of being infected outside Wuhan still exists but the probability is low. Note this issue also applies to the study of Backer et al, Linton et al and Stephen et al, and can be worse as the collected data in their studies were not justified if the assumption is satisfied.

To ensure that the follow-up time is long enough such that no additional biased sampling occurred in this study, we excluded all cases who left Wuhan after January 23, 2020, which leaves an average follow-up time of 25 days (from date of departure to February 15, which is the end of this study). A 25-day follow-up period should be long enough based on the various studies on the incubation period of COVID-19 (3-7). Note that those who left Wuhan after January 23 might not have enough time to develop symptoms before the end of the follow-up period. Including these cases in the cohort might lead to a downward bias on the incubation period. Note that the latest date of symptom onset in our cohort is February 12, 2020, which is three days before the end of the follow-up period. This period should be long enough for a case to develop symptoms. Furthermore, there were only 49 cases who left Wuhan after the lockdown of Wuhan city on January 23, 2020 (27). After examining the collected data, there were a total of 1084 cases that meet the criteria and were followed forwardly.

Table S1 and Figure S1 show the locations of diagnosis and durations between symptoms onset to diagnosis in the Wuhan departure cohort and the entire data collected as of February 15, 2020.
[Table S1]
[Figure S1]

S3

Let Y be the incubation period of an infected case with the probability density function $f(y)$ where $y > 0$. Let A be the truncated time calculated from infection in Wuhan to the departure of Wuhan with $u(\mathrm{a})$ as the corresponding probability density function where $a > 0$. Note in a renewal process A can be considered as the backward time. Let V denote the duration between departure from Wuhan and onset of symptoms, which can be considered as the forward time in a renewal process. Clearly, A is not observable. It is known that in the cross-sectional sampling, $A + V$ is a length-biased version of the incubation period Y, as probability that an interval is selected is proportional to the length of the interval, namely it is easier to observe V if $A + V$ is longer, and hence the mean value $\mathrm{E}(A + V)$ is longer than the

mean incubation period $\mathrm{E}(Y)$. If we can observe Y without taking this sampling bias into consideration, then definitely we overestimate the average incubation period, and the corresponding density is

$$Y|Y > A \sim \frac{U(y)f(y)}{\int_0^\infty U(y)f(y)\mathrm{d}y}, \qquad y \geq 0,$$

where $U(\cdot)$ is the cdf of A. As Y is usually not observable, instead, we can observe the forward time V. Again, the sampling bias still exists, the observed V has density as follows,

$$V = Y - A|Y > A \sim \frac{P(Y - A = v)}{P(Y > A)} = \frac{\int_0^\infty f(a+v)u(a)\mathrm{d}a}{\int_0^\infty \bar{F}(a)u(a)\mathrm{d}a}, \qquad v \geq 0, \tag{S1}$$

and the sampling biased A has density as follows,

$$A|Y > A \sim \frac{P(Y > A|A = a)P(A = a)}{P(Y > A)} = \frac{\bar{F}(a)u(a)}{\int_0^\infty \bar{F}(a)u(a)\mathrm{d}a}, \qquad a \geq 0,$$

where $\bar{F}(\cdot)$ is the survival function of $f(\cdot)$, a and v are the realizations of A and V. In the length bias sampling, the choice of $u(\cdot)$ is a uniform density in $(0, \tau)$ where τ is a fixed large number (25). In COVID-19 example, a possible choice of τ can be 30 days. Under above assumptions,

$$V|Y > A \sim \frac{\bar{F}(v)}{\mu} \equiv g(v), \;\; 0 \leq v \leq \tau, \tag{S2}$$

where $\mu = \int_0^\infty yf(y)\,dy$ is the mean incubation period. Furthermore,

$$A|Y > A \sim \frac{\bar{F}(a)}{\mu} \equiv g(a), \qquad 0 \leq a \leq \tau.$$

Hence, marginally A and V have the same density. More technical detail in regard to the renewal process can be found in Chapter 2 of Qin (25). It is arguable that as the number of infections grew exponentially at the beginning of the epidemic, the uniform assumption of the backward time might be unrealistic. However, the sensitivity analysis in Supplement S4 indicates that equation (S2) is still valid in our studying cohort. See Supplement S4 for more details.

In our cohort of COVID-19 cases, we assume the incubation period is a Weibull random variable with probability density function

$$f(y) = \alpha\lambda(y\lambda)^{\alpha-1}exp\{-(y\lambda)^\alpha\}, \qquad y \geq 0. \tag{S3}$$

Using equations (S2) and (S3), it can be shown that the forward time has the density function as follows

$$g(v) = \alpha\lambda\frac{exp\{-(v\lambda)^\alpha\}}{\Gamma(1/\alpha)}, \qquad v \geq 0. \tag{S4}$$

Let v_i be the observed forward times, $i = 1,2,\dots,I$, where $I = 1084$ in the study, the estimates $\hat{\alpha}$ and $\hat{\lambda}$ can be obtained by maximizing the likelihood function

$$L(\alpha,\lambda) = \prod_{i=1}^{I} \alpha\lambda \frac{exp\{-(\lambda v_i)^\alpha\}}{\Gamma(1/\alpha)}, \qquad \alpha > 0, \lambda > 0. \tag{S5}$$

The mean and percentiles of the incubation period can be calculated from the parametric Weibull distribution. The confidence intervals in this study are obtained using bootstrap method with $\mathrm{B} = 1000$ resamples.

S4

In renewal process, it is common to assume that backward time A is uniformly distributed (25). However as the number of infections grew exponentially at the beginning of the epidemic, it is more likely to observe someone who was infected closer to departure date. In other word, it might potentially contradict the uniform assumption of the backward time A and the equation (S2) might not apply. To overcome this issue we conducted a sensitivity analysis by assuming the distribution of A is an exponential distribution, namely $u(a) = \theta e^{-\theta a},\ \theta \geq 0,\ a \geq 0$. Note that such distribution satisfies the guess that infection occurred closer to departure date. Substituting $u(a)$ into the equation (S1), and let $f(\cdot)$ to be a Gamma density function as an example, namely $f(y) = \lambda^\alpha y^{\alpha-1} e^{-\lambda t}/\Gamma(\alpha)$, we obtain

$$V|Y > A \sim \frac{c\theta}{1-c} e^{v\theta} \bar{F}\left(v; \alpha, \frac{1}{\lambda+\theta}\right), \qquad v > 0, \tag{S6}$$

where $c = \left(\lambda/_{\lambda+\theta}\right)^\alpha$ and $\bar{F}$ is the survival function of Gamma distribution. The maximum likelihood estimates (MLE) were $\hat{\alpha} = 4.66,\ \hat{\lambda}$ =0.52 and $\hat{\theta}$ =7.93× 10^{-8}. Note that if $\theta = 0$, equation (S6) can be simplify to equation (S2). Hence, based on the MLE of $\hat{\theta}$ =7.93× 10^{-8}, we are confident that equation (S2) is valid even without uniform assumption. Note that is if the incubation period follows a Weibull or lognormal distribution, the close form of the density function of V is not available, but the parameters can be still estimated using iterative numerical approximation.

Table S1. Locations of diagnosis in the studying cohort and all cases collected as of February 15, 2020.

Location	Studying cohort	All cases	Location	Studying cohort	All cases
GUANGDONG	117	1316	SHAANXI	33	233
HENAN	260	1232	YUNNAN	14	169
ZHEJIANG	169	1179	HAINAN	2	162
HUNAN	26	1004	GUIZHOU	7	144
ANHUI	113	963	JIANGXI	6	129
JIANGXI	11	926	TIANJIN	8	122
OVERSEAS	27	615	LIAONING	9	120
JIANGSU	47	617	GANSU	4	91
CHONGQING	0	544	JINING	2	89
SHANGDONG	68	537	NINGXIA	4	71
SICHUAN	69	481	XINJAING	2	71
HEILONGJIANG	1	460	INNER MONGOLIA	2	70
BEIJING	1	383	HONG KONG	3	36
SHANGHAI	0	328	QINGHAI	1	18
HEBEI	21	300	TAIWAN	1	18
FUJIAN	2	287	MACAO	1	10
GUANGXI	53	237	TIBET	0	1

Figure S1. Histogram of duration from symptoms onset to positive diagnosis in the studying cohort and all cases collected as of February 15, 2020. The mean duration is 4.62 days and 5.723 days in the studying cohort and all cases respectively which might indicate that patients with travel or residency history at Wuhan are more self-awareness about COVID-19.

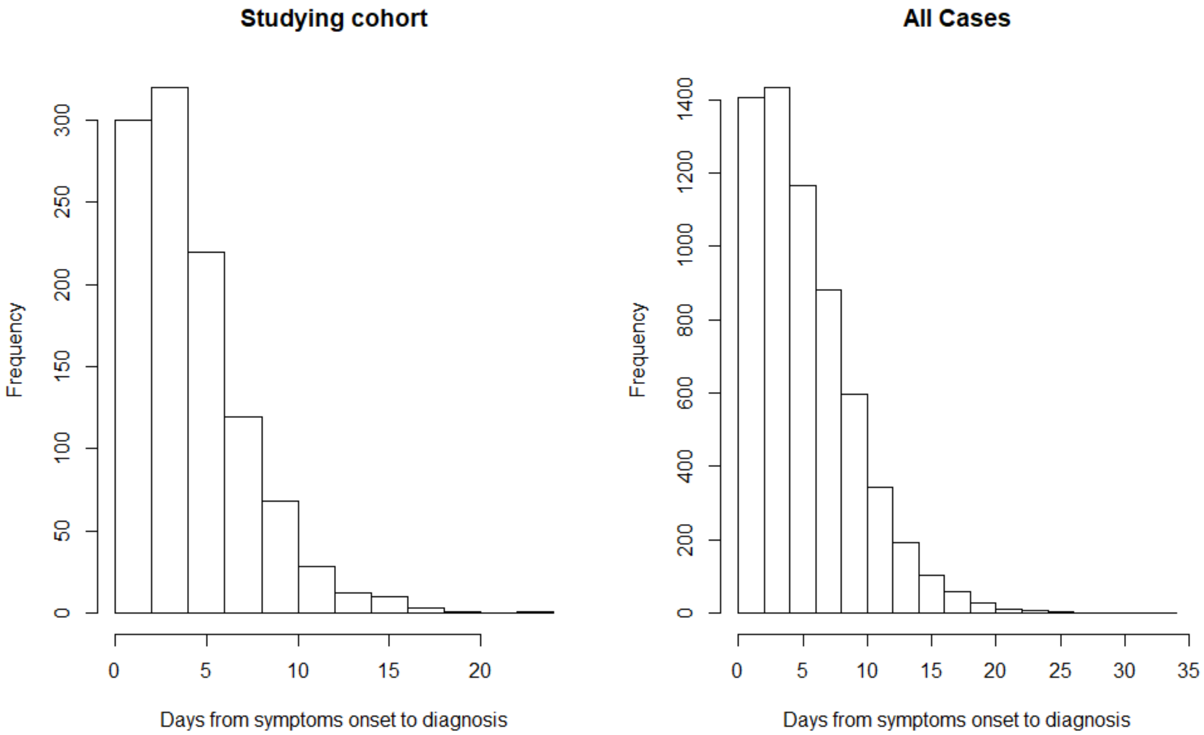

Supplement: abc1202_SM.pdf [file abc1202_SM.pdf]
